# Bilateral Mydriasis in a Post-parotidectomy Patient: A Case Report

**DOI:** 10.34172/aim.34254

**Published:** 2025-06-01

**Authors:** Ahmad Daneshi, Saleh Mohebbi, Milad Shemshadi, Hadi Ghanbari

**Affiliations:** ^1^Head & Neck Research Department, Hazrat Rasoul Hospital, Iran University of Medical Sciences, Tehran, Iran

**Keywords:** Case report, General anesthesia, Myasthenia gravis, Mydriasis

## Abstract

Anticholinergic agents, such as atropine and glycopyrrolate, are commonly utilized during anesthesia for their effects on secretion reduction and vagal activity. However, substantial dosages can induce mydriasis, which poses diagnostic challenges, particularly in head and neck surgeries. Despite their clinical relevance, limited studies explore these effects. A 35-year-old female presented with a left parotid mass and scheduled for a left superficial parotidectomy. Preoperatively, the patient exhibited normal ocular and neurological function. Postoperatively, fine bilateral ptosis, predominantly on the left side, and bilateral unresponsive mydriasis were noted. Anticholinergic-induced pupillary changes may mimic neurological pathology, underscoring the necessity for meticulous postoperative evaluation and awareness among clinicians.

## Introduction

 Sympathomimetic drugs containing local anesthetics or systemic anticholinergics are frequently used in different conditions and can cause unilateral or bilateral pupillary dilatation.^1^ Evidence suggests that the sympathetic nervous system does not mediate pupillary dilatation.^2^ anticholinergic drugs were widely used as a premedication in general anesthesia in order to reduce the secretion and vagal over activity and prevent bradycardia during the surgery. In addition, these drugs were used at the time of reversal of neuromuscular block.^3^ The administration of anticholinergic agents, particularly atropine and glycopyrrolate, at substantial dosages induces pupillary dilation (mydriasis) in adult patients who present with normal ocular function, with strabismus being the sole exception. This pharmacological response occurs through the blockade of parasympathetic nerve signals at muscarinic receptors in the iris sphincter muscle. It is noteworthy that when these medications are administered at standard therapeutic doses via intramuscular or intravenous routes, they typically do not elicit significant mydriatic effects. This dose-dependent relationship between anticholinergic drugs and pupillary response serves as an important clinical consideration in both anesthetic practice and emergency medicine.^4^ there is a diagnostic challenge due to the mydriatic effects of these drugs can interfere the neurological assessment after surgery. Despite their widespread use, there is limited studies exploring this side effect of anticholinergic-induced pupillary changes in surgeries, specifically in head and neck surgeries.

## Case Report

**Figure 1:**
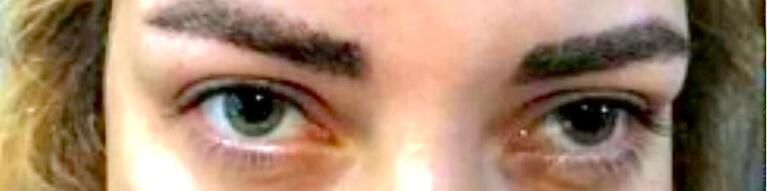
Bilateral Mydriasis of Unknown Etiology After General Anesthesia for Left-Side Parotid Mass Resection

 The case was a 35-year-old female with no significant previous medical history that referred to our hospital clinic with the chief complaint of a left parotid mass that she noticed from 2 years ago, gradually increasing in size without any pain or skin involvement. In physical examination, she was a young female with lightly pigmented eyes and an asymmetrical face in the parotid area. A mass was palpated in the left parotid with a diameter of 2.5 cm × 3 cm with no sign of skin involvement or tenderness. The facial nerve examination was normal and symmetrical. She had done a CT-scan and MRI before, and parotid pleomorphic adenoma was confirmed by fine needle aspiration cytology (FNAC). According to this set of data, she was scheduled for left superficial parotidectomy. In the COVID-19 era, RT-PCR for COVID-19 was negative, and other laboratory tests were within normal limits (Table 1). In the operating room, lidocaine and epinephrine 1/100 000 were locally administered before skin incision. At the start of induction, total intravenous anesthesia was administered using thiopental 400 mg, atracurium 35 mg, fentanyl 15 mg, midazolam 2 mg, remifentanil 200 microgram, propofol 1% (300-400 mg total) during the operation time of approximately 70 min. The patient’s condition was reversed by atropine 1.5 mg and neostigmine 3 mg. The patient awakened slowly with no problem, but slower than the other patients with no facial nerve dysfunction in the recovery room. The patient received no antibiotic or other drugs locally or systemically in the operating room or after that. While visiting the patient post-operation in the ward, the senior surgeon noted a fine, barely detectable bilateral ptosis, predominantly on the left side. Recognizing bilateral unresponsive mydriasis in both eyes (Figure 1) and a quick overshooting upward movement followed by a downdrift of the upper lid was denoted as a positive Cogan’s sign. According to these findings, neurological disorders such as myasthenia gravis were suspected, and following imaging including Magnetic resonance imaging revealed no abnormal findings in the brain or brainstem, and pulmonary CT-scan was unremarkable. Acetylcholine receptor (AChR) antibody and COVID-19 serologic tests were requested. Immunological tests of COVID-19 (IgG and IgM) were negative, and the AChR antibody was positive, and the myasthenia gravis was confirmed (Table 2). In this case, we could consider the mydriasis as a side effect of the anti-cholinergic drugs used during general anesthesia, but precise examination and broader investigations helped us to detect the disease in its very early stage.

**Table 1:** Baseline Preoperative Laboratory Values

**Test**	**Result**	**Unit**	**Reference Value**
Hb	12.8	g/dl	Female: 12-16
Plt	226	^*^1000/mm3	140-440
Blood Sugar	96	mg/dL	70-115
B.U.N	13	mg/dL	5-23
Creatinine	0.8	mg/dL	0.5-1.5
INR	1.08	Index	1-1.1
PTT	31	Sec	25-40

**Table 2:** Serological Test Result Supporting the Diagnosis of Myasthenia Gravis

**Test**	**Result**	**Unit**	**Reference Value**
AChR antibody	0.7	Nmol/L	Negative < 0.4, Borderline > 0.4- < 0.5, Positive > 0.5

## Discussion

 Large doses of anticholinergic drugs (atropine, glycopyrrolate) produced mydriasis, though the usual intramuscular and intravenous doses of these drugs do not have this tendency. Neostigmine methyl-sulfate reduces the mydriatic effect when given intravenously in conjunction with atropine. Mydriasis was more likely to occur in lightly pigmented eyes.^3^ A 35-year-old female with a lightly pigmented eye was admitted to an outpatient clinic for parotidectomy. A meticulous history was taken from the patient, revealing the routine daily intake of propranolol 20 mg/d and levothyroxine 100 mg/d early in the morning of the operation day. No potential mydriatic effects were recognized before the injection of reverse drugs as a combination of atropine and Neostigmine. Volatile anesthesia was not used as isoflurane or sevoflurane, and bilateral mydriasis regression was delayed over 24 hours. Our case is not the same as that demonstrated utilizing inhalational drugs. could induce mydriasis^1,5,6^ There were no anticholinergic effects on increasing cardiac output or heart rate in our patient.^7^ Although patients with potentially increased intracranial pressure and mydriasis are often first investigated in the emergency room or intensive care unit, it is crucial to know the drugs that can be used as volatile anesthetics that may be able to induce mydriasis under exceptional circumstances. The patient was in good condition and had no symptoms of systemic atropine poisoning such as drowsiness, central nervous system depression, circulatory collapse with respiratory failure, sudden dizziness, headache, confusion, balance problems, or other symptoms of a possible stroke.^8^ Although Cogan’s lid twitch is not specific for ocular myasthenia gravis, but it should be considered.^9^ In our study, we faced a patient whose AChR antibody was more than the range, denotes myasthenia gravis with undetectable ptosis; Cogan’s positive sign and ocular presentation confirm the disease with the highest specificity 100%,^10^ which is rewarding for the patient and physician before presenting symptoms of the actual disease occur.

## Conclusion

 This case highlights the diagnostic complexity associated with bilateral mydriasis following anticholinergic use during general anesthesia, particularly in patients with lightly pigmented eyes. The persistence of mydriasis beyond the immediate postoperative period and the presence of a positive Cogan’s sign prompted a broader diagnostic evaluation, leading to the identification of ocular myasthenia gravis with high specificity. This underscores the importance of recognizing subtle ocular signs as potential indicators of underlying neuromuscular conditions, which could be critical for early diagnosis and management. Clinicians should maintain a high index of suspicion for non-neurological causes of mydriasis while also considering systemic implications, especially during routine anesthetic practice.
